# Atypical Inguinal Hernia: Sonography for Fat Herniation Through the Canal Wall Defect

**DOI:** 10.7759/cureus.72832

**Published:** 2024-11-01

**Authors:** Kanyamas Poompreecha, Wei-Ting Wu, Ke-Vin Chang, Levent Ozcakar

**Affiliations:** 1 Rehabilitation Medicine, Charoenkrung Pracharak Hospital, Bangkok Metropolitan Administration, Bangkok, THA; 2 Physical Medicine and Rehabilitation, National Taiwan University Hospital Bei-Hu Branch, Taipei, TWN; 3 Physical and Rehabilitation Medicine, Hacettepe University Medical School, Ankara, TUR

**Keywords:** groin, hernia, mass, ultrasonography, ultrasound

## Abstract

A groin mass can arise from multiple conditions, with common causes including hernias, enlarged lymph nodes, fluid collections, or tumors. An ultrasound, together with a clinical examination, plays a key role in diagnosing groin masses and identifying hidden hernias. A 44-year-old male presented with a painless right inguinal mass that had been continuously present for one month. Upon clinical examination, a positive silk-glove sign was noted, though both static and dynamic ultrasound imaging did not reveal the typical characteristics of hernias. Instead, a fat-containing mass, measuring 20.6 × 6.4 × 5.0 mm, was observed through a small defect in the inguinal canal wall near the superficial inguinal ring, without any vascular or tubular involvement. Due to minimal symptoms, the patient was advised to undergo conservative treatment, with a diagnosis of peritoneal fat herniation. This case emphasizes the value of high-resolution ultrasound in evaluating groin masses and suggests that in the absence of a typical hernia sac, conservative management and monitoring may help avoid unnecessary surgery.

## Introduction

Groin masses are frequently encountered in clinical practice and can originate from a wide array of causes. Common etiologies include lymphadenopathy, infections, fluid collections, and tumors, each with its distinct clinical implications. Among these, inguinal hernias are particularly significant, accounting for approximately 75% of all abdominal wall herniations, making them one of the most common surgical diagnoses [1]. While many groin masses can be identified based on clinical examination alone, such as the Valsalva maneuver, the complexity of certain cases often requires the use of advanced imaging techniques to provide a more accurate diagnosis.

Ultrasound, computed tomography (CT), and magnetic resonance imaging (MRI) are frequently employed to evaluate groin masses. Each modality has its strengths, but ultrasound stands out for its unique combination of benefits, including low cost, real-time imaging, and the ability to avoid radiation exposure. It is particularly valuable for assessing hernias, where dynamic evaluation during maneuvers such as the Valsalva can help reveal intermittent protrusions that might otherwise go unnoticed [2-4]. Moreover, ultrasound can offer detailed anatomical information about the contents of a mass, enabling clinicians to distinguish between different types of hernias, as well as between hernias and other pathologies such as lipomas or lymphadenopathy [5].

Inguinal hernias typically present with characteristic features on ultrasound such as a protrusion of bowel or fat through the inguinal canal [4]. However, not all groin masses follow this classic presentation, and atypical cases can pose a diagnostic challenge. In some instances, a mass may not exhibit the expected findings on imaging, requiring a deeper investigation and a more comprehensive differential diagnosis. Fat herniation through the inguinal canal, for example, is a less common but clinically important condition that may not exhibit the classic signs of a hernia in imaging studies.

In this report, we present a unique case of a groin mass in a 44-year-old male, where an ultrasound examination revealed fat herniation through a defect in the inguinal canal wall. This case underscores the importance of considering less common diagnoses in patients with groin masses and highlights the utility of high-resolution ultrasound in identifying subtle anatomical abnormalities that may be missed during clinical examination.

## Case presentation

A 44-year-old male presented with a painless right inguinal mass for the last month. He worked as a sports equipment salesperson and was regularly engaged in physical activities such as running, cycling, and weight training. He also reported a family history of hernia in his father and grandfather. The patient had no underlying diseases or history of chronic constipation. His body mass index was 19.7 kg/m², and he had a well-proportioned body shape. Physical examination involving palpating the inguinal canal against the pubic tubercle revealed a positive silk-glove sign.

**Figure 1:**
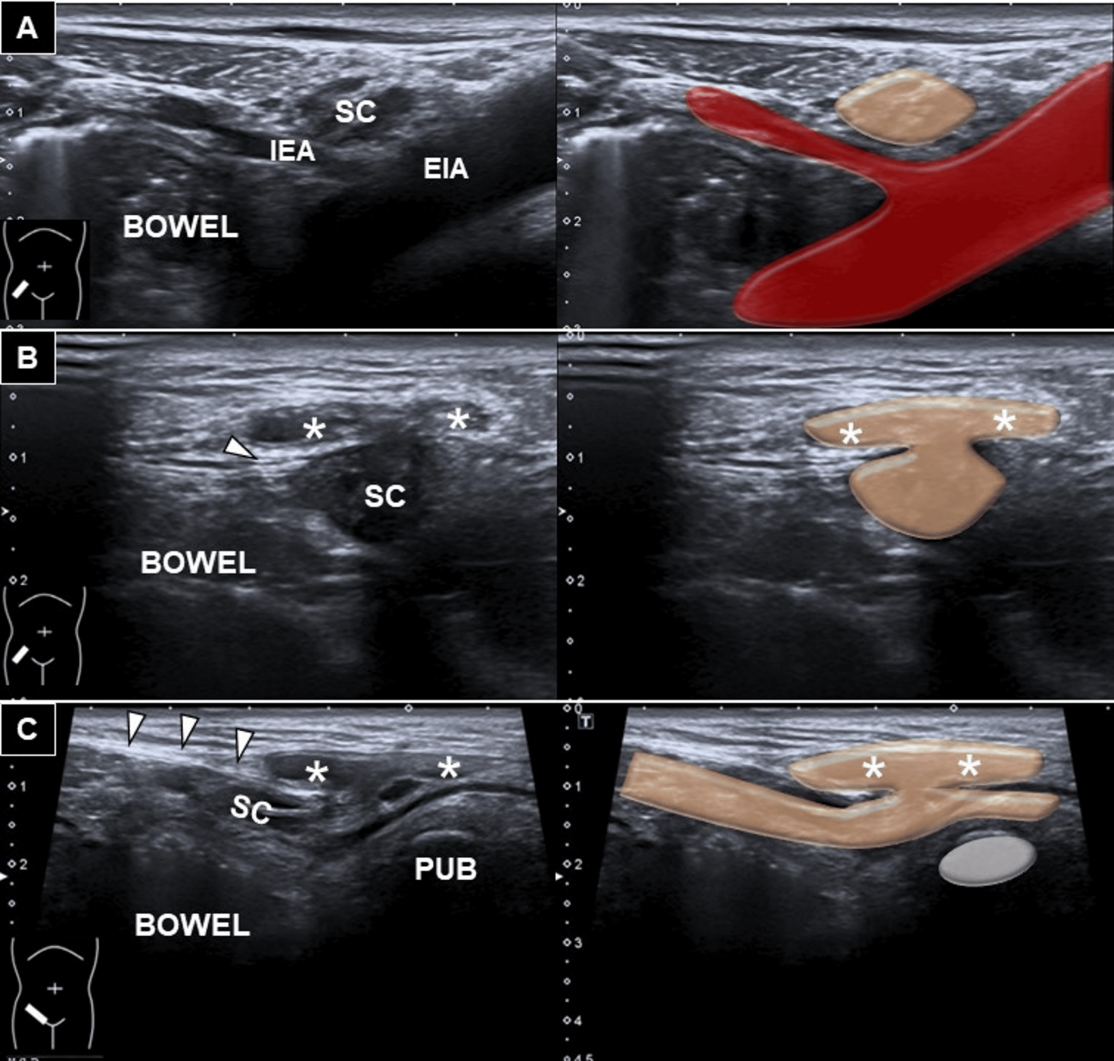
Sonography for fat herniation through the canal wall defect Ultrasound imaging and schematic illustration of (A) the normal architecture at the deep inguinal ring in the short-axis view. As the transducer is moved along the inguinal canal toward the superficial inguinal ring, protrusion of a hypoechoic mass through the wall defect is observed in short- (B) and long-axis (C) views. White arrowhead: inguinal ligament, asterisk: mass, SC: spermatic cord, IEA: inferior epigastric artery, EIA: external iliac artery, PUB: pubic bone

An ultrasound examination using a high-frequency linear transducer (13-18 MHz; Aplio 500, Canon Medical Systems Europe B.V., Netherlands) was performed to confirm the diagnosis. Static and dynamic imaging of the deep inguinal ring showed no evidence of bowel content or internal organs sliding through the inguinal canal or Hesselbach’s triangle. However, a 20.6 × 6.4 × 5.0 mm fat-containing mass was observed protruding from the inguinal canal into the subcutaneous tissue through a defect near the superficial inguinal ring (Figure 1 and Video 1). No anechoic lesions were detected along the inguinal canal or within the scrotal sac during transducer tracing. Dynamic Valsalva maneuver demonstrated no reducible movement of the observed mass. Given the minor symptoms and the diagnosis of fat herniation through an inguinal canal wall defect, the patient was advised to pursue conservative management with local compression.

**Video 1:** Sonography for fat herniation through the canal wall defect The video demonstrates the protrusion of a hypoechoic mass through the wall defect at the superficial inguinal ring, shown in both short-axis and long-axis views. Black arrowhead: superficial inguinal ring, asterisk: mass, PUB: pubic bone

## Discussion

Abdominal wall hernias are seen with a prevalence of 1.7% across all age groups [1]. The internal tissue, such as the intestine, fat, or other organs, is pushed through the weak area of the abdominal wall, creating a noticeable bulge or discomfort, remarkable when increasing the abdominal pressure during lifting heavy objects, coughing, or physical exertion. The most common type is an inguinal hernia, with a lifetime risk of 27% in men and 3% in women [2].

While imaging with ultrasonography, identifying the inferior epigastric artery is crucial for differentiating between indirect and direct inguinal hernias. The deep inguinal ring is lateral to the junction between the inferior epigastric artery and the external iliac artery [6]. In indirect inguinal hernias, the hernial sac protrudes through the deep inguinal ring to the inguinal canal. In contrast, direct inguinal hernias protrude through Hesselbach's triangle, the space between the lateral border of the rectus abdominis muscle and the inferior epigastric artery [5,6]. Another type of inguinal hernia is a femoral hernia, which is located below the inguinal ligament and protrudes through the femoral canal via the femoral ring. During imaging, the transducer is placed lateral/caudal to the inguinal ligament near the pubic tubercle. The femoral artery and vein can be visualized as they pass beneath/superficial to the pubic ramus. The femoral ring is identified as the area medial to the femoral vein [5].

Our case represents an uncommon finding not previously described in the literature, where no hernia sac protrudes through the deep inguinal ring, Hesselbach’s triangle, or femoral ring. However, when the transducer was traced down to the superficial inguinal ring, which is cranial-lateral to the superior pubic tubercle, a defect in the anterior wall of the inguinal canal was observed, with a fat and fluid-containing mass extending into the subcutaneous layer. Although this condition resembles a muscle hernia, it differs in content, i.e. fat and a small amount of intra-canal fluid. Muscle hernias are commonly seen in the lower limbs [7], whereby part of the muscle tissue protrudes through a defect/tear in the fascia. Fortunately, dynamic ultrasound examination confirmed no tubular and significant vascular structures such as vas deferens and testicular vessels herniating into the mass.

Herein, another possible condition could be a spermatic cord lipoma, which is commonly found during inguinal hernia repair. In clinical settings, lipoma without an inguinal hernia sac occurs in 1%-8% of the cases [8]. They typically appear as a hyperechoic mass adjacent to the spermatic cord fascia but not connected with the abdominal fat. Because a spermatic cord lipoma can resemble a primary hernia and has the potential to recur or become symptomatic later, close follow-up is recommended, with possible/future need for surgical excision [8].

## Conclusions

This case report discusses the uncommon condition of fat herniation through an inguinal canal wall defect as part of the differential diagnosis for a groin mass. Ultrasonography plays a critical role in diagnosing the mass, determining its size, and assessing its anatomical relevance and content, facilitating more effective management planning for these rare cases.

## References

[1] Jenkins JT, O'Dwyer PJ (2008). Inguinal hernias. BMJ.

[2] Kingsnorth A, LeBlanc K (2003). Hernias: inguinal and incisional. Lancet.

[3] Shadbolt CL, Heinze SB, Dietrich RB (2001). Imaging of groin masses: inguinal anatomy and pathologic conditions revisited. Radiographics.

[4] Lee RK, Cho CC, Tong CS, Ng AW, Liu EK, Griffith JF (2013). Ultrasound of the abdominal wall and groin. Can Assoc Radiol J.

[5] Picasso R, Pistoia F, Zaottini F (2021). High-resolution ultrasound of spigelian and groin hernias: a closer look at fascial architecture and aponeurotic passageways. J Ultrason.

[6] Wu WT, Chang KV, Lin CP, Yeh CC, Özçakar L (2022). Ultrasound imaging for inguinal hernia: a pictorial review. Ultrasonography.

[7] Nguyen JT, Nguyen JL, Wheatley MJ, Nguyen TA (2013). Muscle hernias of the leg: a case report and comprehensive review of the literature. Can J Plast Surg.

[8] Köckerling F, Schug-Pass C (2020). Spermatic cord lipoma--a review of the literature. Front Surg.

